# Overexpression of Cassava *MeAnn2* Enhances the Salt and IAA Tolerance of Transgenic *Arabidopsis*

**DOI:** 10.3390/plants10050941

**Published:** 2021-05-08

**Authors:** Xuejun Lin, Ruimei Li, Yangjiao Zhou, Fenlian Tang, Yajie Wang, Xiaohua Lu, Shijia Wang, Yuan Yao, Jiao Liu, Xinwen Hu, Jianchun Guo

**Affiliations:** 1 College of Tropical Crops, Hainan University, Haikou 570228, China; 18095131210044@hainanu.edu.cn (X.L.); liruimei@itbb.org.cn (R.L.); 18071010110007@hainanu.edu.cn (Y.Z.); 19095131210051@hainanu.edu.cn (F.T.); wyj5501@hainanu.end.cn (Y.W.); 19071010110006@hainanu.edu.cn (X.L.); 20086000210042@hainanu.edu.cn (S.W.); huxinwen@hainu.edu.cn (X.H.); 2 Institute of Tropical Bioscience and Biotechnology, Chinese Academy of Tropical Agricultural Sciences, Haikou 571101, China; yaoyuan@itbb.org.cn (Y.Y.); liujiao@itbb.org.cn (J.L.)

**Keywords:** *Manihot esculenta*, annexin, *MeAnn2*, *Arabidopsis thaliana*, genetic transformation, stress analysis

## Abstract

Annexins are a superfamily of soluble calcium-dependent phospholipid-binding proteins that have considerable regulatory effects in plants, especially in response to adversity and stress. The *Arabidopsis thaliana AtAnn1* gene has been reported to play a significant role in various abiotic stress responses. In our study, the cDNA of an annexin gene highly similar to *AtAnn1* was isolated from the cassava genome and named *MeAnn2*. It contains domains specific to annexins, including four annexin repeat sequences (I–IV), a Ca^2+^-binding sequence, Ca^2+^-independent membrane-binding-related tryptophan residues, and a salt bridge-related domain. *MeAnn2* is localized in the cell membrane and cytoplasm, and it was found to be preferentially expressed in the storage roots of cassava. The overexpression of *MeAnn2* reduced the sensitivity of transgenic *Arabidopsis* to various Ca^2+^, NaCl, and indole-3-acetic acid (IAA) concentrations. The expression of the stress resistance-related gene *AtRD29B* and auxin signaling pathway-related genes *AtIAA4* and *AtLBD18* in transgenic *Arabidopsis* was significantly increased under salt stress, while the Malondialdehyde (MDA) content was significantly lower than that of the control. These results indicate that the *MeAnn2* gene may increase the salt tolerance of transgenic *Arabidopsis* via the IAA signaling pathway.

## 1. Introduction

Annexins, similar to calmodulins, are multifunctional phospholipid-binding proteins that bind calcium ions and phospholipids in the membrane in response to external stimuli. Studies have shown that plant annexins are widely present in plant cells and have roles in plant growth, development, and environmental responses, amongst other processes. The structures of plant annexins are evolutionally conserved, whereby the N-terminal domain of each annexin is variable, while the C-terminal core domain is conserved. The N-terminal, as a variable region, may have different amino acid sequence lengths in each annexin, containing multiple protein-binding sites, such as for proteolysis and phosphorylation. Therefore, the N-terminal functionally serves as the regulatory region of the annexin molecule and the basis by which members of different annexin families can be distinguished [1,2,3,4]. The conserved C-terminal homologous domains are generally composed of four highly similar repeats of amino acids (Repeats I–IV), each containing about 70 amino acid residues. Generally, only Repeat I and Repeat IV contain the K–G–X–G–T–(38)–D/E motif, which is the type II Ca^2+^-binding site [1,3], while Repeat II and III do not have the above motif [1,3,4]. Each repeat has five α-helical folding (helix A–E) secondary structures, which are closely stacked to form a disk-like structure with a convex side and a concave side [3,4]. Additionally, the membrane binding sites of each repeat are located in the helix A-B and helix D-E loops which are belong to the convex side of the disk structure [3]. The type II Ca^2+^-binding site G–X–G–T locates in the helix A-B loops, while the bidentate acidic side chain is found in the helix D [3]. In plant cells, the convex side of annexin is oriented toward the biofilm and the concave side is oriented toward the cytoplasm when the annexin as mediated by Ca^2+^, and the N-terminal domain can interact with other proteins or molecules in the cytoplasm as part of the annexin function [1,3,4]. The localization of annexin in cells is the key factor in its various functions. At present, most of the known plant annexins are mainly located in the cytoplasm, while some are distributed in the plasma membrane, organelle membranes, nuclear membrane, and nucleoli [5]. For example, in *Medicago sativa, MtAnn1* is located on the nuclear membrane [6]. Annexin in *Pisum sativum* is localized in nucleoli [7]. The subcellular localization of plant annexin is related to the cytoplasmic Ca^2+^ concentration, cell environment pH, plant tissues, and external environment. In addition, annexin can be transferred within plants under different stress environments [8], and the expression of annexin in tissues varies greatly with the growth and development of plants [9,10,11,12,13]. This demonstrates that annexins, as functional proteins, are involved in the regulation of plant metabolism, growth, and development. The structure and localization of plant annexins determine their functions in plant growth, cell secretion, Ca^2+^ metabolism, and regulation of abiotic stress. For example, the observation and analysis of transgenic *Arabidopsis* roots successfully transfected using annexin tagged with green fluorescent protein (GFP) revealed that annexin could promote Ca^2+^ influx in *Arabidopsis* protoplasts, thereby increasing intracellular Ca^2+^ concentrations [14,15,16]. 

Soil salinization is one of the main environmental factors hampering crop growth and reducing productivity [17]. Under salt stress, a series of molecular chemical reactions take place in plants. The mechanisms are very complex and are regulated and involved by a variety of genes. At the early stage of salt stress, the signal of the salt stress response involves the increase in cytoplasmic Ca^2+^ concentration [18,19]. Ca^2+^ waves were found to spread through the root system after 10 s of salt stress treatment and reached the leaves within 30 s [20]. Many researchers have revealed the important role of plant annexins in salt stress tolerance in plants. For example, studies have shown that there are eight *AtAnn* genes in the *Arabidopsis* genome, the expression of *AtAnn1,* and *AtAnn8* was significantly affected by salt stress induction [15,21]. *AtAnn4* has been shown to mediate salt stress-induced Ca^2+^ increase, which is related to salt-specific calcium signaling production and transmission [22]. The *AtAnn1* and *AtAnn4* can interact with each other and contribute to the salt resistance [23]. Separate mutations *AtAnn1* and *AtAnn4*, respectively, led to increased salt sensitivity in *Arabidopsis* [8]. In Indian mustard (*Brassica juncea*), six annexin genes were cloned and characterized [24]. Tobacco seedlings overexpressing *BjAnn1* or *BjAnn2* are more resistant to salt damage [25,26]. In durum wheat (*Triticum durum*), the *TdAnn6* and *TdAnn12* were spatially regulated under salt stress and other stress [27]. Overexpression of the *TdAnn6* gene in *Arabidopsis* increased the germination rate of transgenic seeds under 150 mM NaCl stress, and significantly increased the root growth and fresh biomass of transgenic seedlings under salt stress [28]. In cotton (*Gossypium hirsutum*), there are 26 putative GhAnns in the genome, and the *GhAnn8b* was found to improve the salt tolerance of the plant by overexpressing it, while, when silencing the *GhAnn8b*, the cotton was more sensitive to salt than the control plants. Additionally, it was found that cotton *GhAnn8b* can be phosphorylated by salt stress induction [29]. 

Developmental plasticity of plant roots is a marker of response to abiotic stress [30]. The effects of salt stress on the main and lateral root development of *Arabidopsis thaliana* at the seedling stage were obvious. The inhibition of root growth under salt stress was related to the decrease in auxin level [31]. In *Arabidopsis*, the genes of LATERAL ORGAN BOUNDARIES DOMAIN (LBD) and INDOLE-3-ACETIC ACID (IAA) were found to contribute to the lateral root emergence [32,33,34]. Recently, it has been reported that *AtLBD18* and *AtIAA4* were significantly induced by salt stress in salt tolerance *Capsicum annuum CaNAC4* transgenic *Arabidopsis* [35]. Overexpressing the *CaNAC4* in *Arabidopsis*, the transgenic plants gain tolerance to salt stress, as their roots grew better than the control plants. Meanwhile, the root growth-related genes such as *AtLBD18* and *AtIAA4* were significantly up-regulated in transgenic plants [35]. 

Cassava (*Manihot esculenta* Crantz) is an important starch crop which has good tolerance to drought and barren soil [36,37]. In this study, we successfully cloned the cDNA sequence of an annexin gene *MeAnn2*, from cassava SC8 (South China No. 8) cultivar. It shares 70.9% amino acid sequence similarity with AtAnn1, and it contains domains specific to annexins. The overexpression of *MeAnn2* reduced the sensitivity of transgenic *Arabidopsis* to various Ca^2+^, NaCl, and IAA concentrations. The expression of the stress resistance-related genes in transgenic *Arabidopsis* was significantly increased under salt stress. These results provide new knowledge of the role of *MeAnn2* gene in plant stress resistance. 

## 2. Results

### 2.1. Cloning and Characterization of MeAnn2 in Cassava

The cDNA of *MeAnn2* (GenBank No. KM975563) was cloned from cassava (Figure S1) based on its sequence homology to *AtAnn1*. The *MeAnn2* coding sequence (CDS) was 951 bp and encodes 316 amino acids. The predicted molecular mass of MeAnn2 protein is 36.05 kDa, and its isoelectric point was 6.34. The hydrophilic index of MeAnn2 protein was calculated as −0.525, indicating that MeAnn2 is a stable hydrophilic protein. The protein instability index of MeAnn2 was calculated as 24.66, indicating that it is a stable protein. 

**Figure 1:**
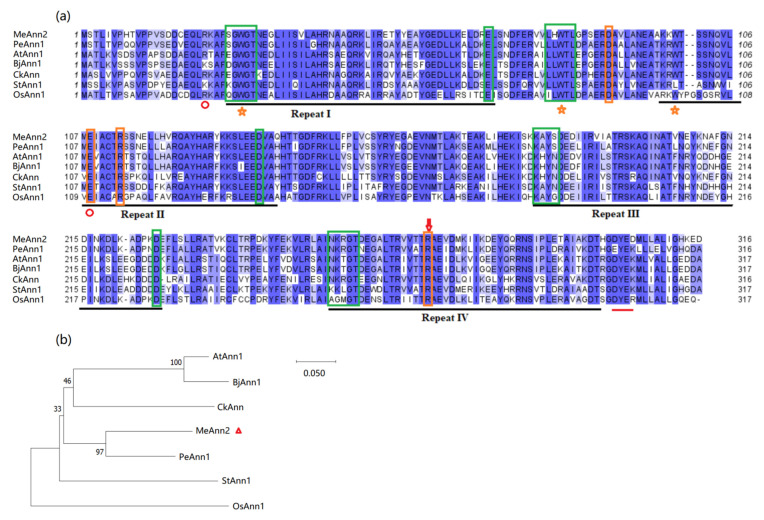
Multiple sequence alignment and phylogenetic relationships of MeAnn2 from cassava with other annexins from different species. (**a**) Multiple sequence alignment. The dark and light blue shadows indicate the amino acid residues with greater than 50% identity. The black line marks the annexin repeat I–IV. The orange star indicates the conserved tryptophan required for Ca^2+^-independent membrane binding. The red line indicates the diacidic motif, an important signal for endoplasmic reticulum export to the plasma membrane. The orange boxes indicate the channel-function-related salt bridges, and the green boxes indicate the Ca^2+^-binding sequences. The red circle indicates the phosphatidylserine-binding motifs. (**b**) Phylogenetic relationships between MeAnn2 (*Manihot esculenta*, KM975563) and annexin proteins from other species. AtAnn1 (*Arabidopsis thaliana*, NP_174810), PeAnn1 (*Populus euphratica*, XP_011016329), OsAnn1 (*Oryza sativa*, XP_015644251), StAnn1 (*Solanum tuberosum*, XP_006355349.1), CkAnn (*Cynanchum komarovii*, GU067483), BjAnn1 (*Brassica juncea*, AAR10457).

Multiple sequence alignment indicated that *MeAnn2* shares 81.9%, 70.9%, 69.0%, 67.7%, and 68.4% identity with *PeAnn1*, *AtAnn1*, *BjAnn1*, *CkAnn*, and *StAnn1*, respectively. All annexins possess four annexin repeats (Repeat I–IV), Ca^2+^-binding sequences, Ca^2+^-independent membrane-binding-related tryptophan residues, channel-function-related salt bridges, endoplasmic-reticulum-export-to-plasma-membrane-related diacidic motifs, and phosphatidylserine-binding motifs (Figure 1a). There are also some changed amino acid residues in the MeAnn2 compared to AtAnn1 and other Anns. For example, the third green box in MeAnn2 is “LHWTL”, while “LLWTL” in PeAnn1, AtAnn1, BjAnn1, CkAnn1 and StAnn1, and “ILWTL” in OsAnn1. The motif of the fifth green box is “KAYS” in MeAnn2 and PeAnn1, but “KHYN” in AtAnn1, BjAnn1 and CkAnn1, and “KAYN” in StAnn1, “KAYG” in OsAnn1. The motif of the seventh green box is “NKRGT” in MeAnn2, PeAnn1 and CkAnn1, but it is “NKTGT” in AtAnn1 and BjAnn1, “KKLGT” in StAnn1, and “AGMGT” in OsAnn1. The evolutionary relationship of MeAnn2 and other annexins shows that its closest protein ortholog is PeAnn1, and it is also closer to CkAnn, AtAnn1, and StAnn1 of dicotyledons while being more distantly related to OsAnn1 of a monocotyledon (Figure 1b).

### 2.2. Subcellular Localization of the MeAnn2 Protein

**Figure 2:**
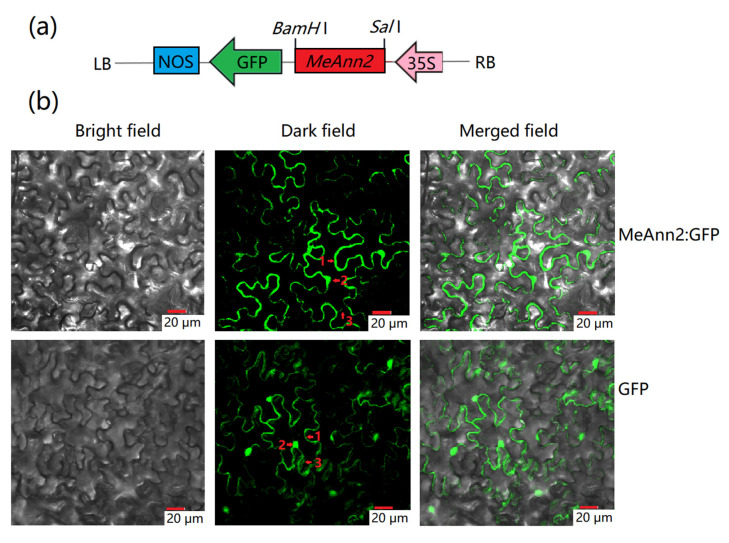
Subcellular localization analysis of the MeAnn2 protein in tobacco leaf cells. (**a**) Schematic diagram of pCAMBIA1300-35S-*MeAnn2:GFP* vector construction; LB indicates the left border and RB indicates the right border of the pCAMBIA1300 vector. (**b**) The fluorescence signal detected in transfected cells. From left to right, the picture is of a bright field, dark field, and merged field of light and shade. The scale is 20 μm. The red arrows and red number point out the localization of MeAnn2. 1, cell membrane; 2, nucleus; and 3, cytoplasm.

To explore the localization of the protein expressed by the *MeAnn2* gene as an indicator of function, a fusion expression vector of pCAMBIA1300-35S-*MeAnn2*:*GFP* was constructed (Figure 2a), and transient expression was mediated by *Agrobacterium tumefaciens* in tobacco leaves. The empty vector pCAMBIA1300-35S-*GFP* was used as the control. After 3–5 days of co-culturing, laser confocal microscopy showed that the fluorescence signal corresponding to *MeAnn2*:*GFP* in transfected tobacco leaf cells could be observed in the cell membrane and cytoplasm. The fluorescence signal in cells transfected with control vector was observed in the cell membrane, cytoplasm, and nucleus (Figure 2b).

### 2.3. Analysis of MeAnn2 Expression in Cassava

**Figure 3:**
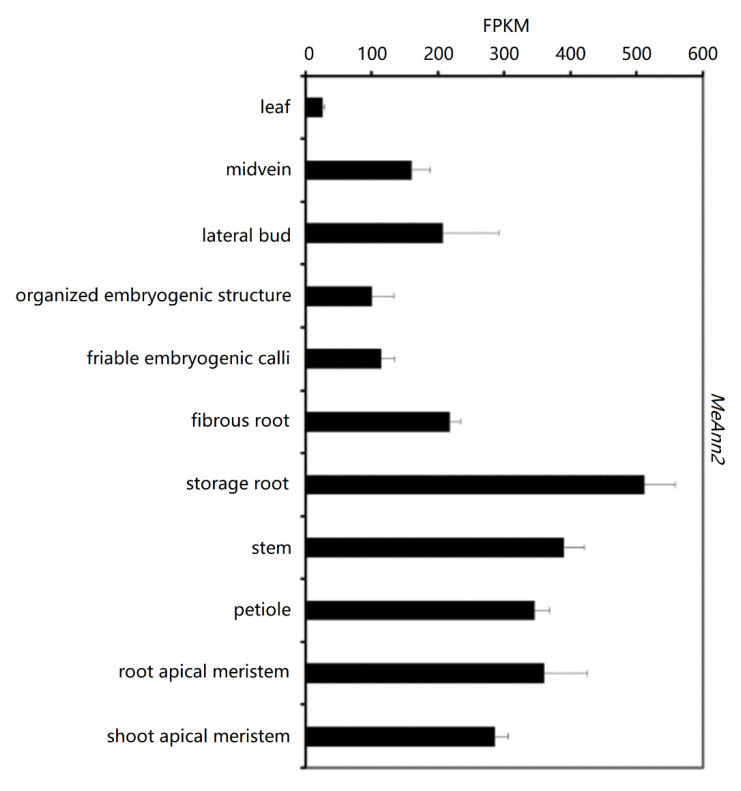
Analysis of *MeAnn2* gene expression in different organs or tissues. Error bars indicate ± SD.

To further study the function of cassava *MeAnn2*, we analyzed the expression patterns in specific cassava tissues based on existing cassava transcriptome data. In the transcriptome data, mRNA was sequenced and quantified in 11 organs/tissues. The *MeAnn2* gene expression data (FPKM values) were picked out to draw a histogram. The results show that the *MeAnn2* gene was expressed in all tested organs/tissues, but the expression levels varied greatly. The expression level of the *MeAnn2* gene was highest in the storage roots, followed by the stems, root apical meristems, petioles, and stem apical meristems, and was the lowest in the leaves (Figure 3). 

### 2.4. Transformation of MeAnn2 in Arabidopsis

**Figure 4:**
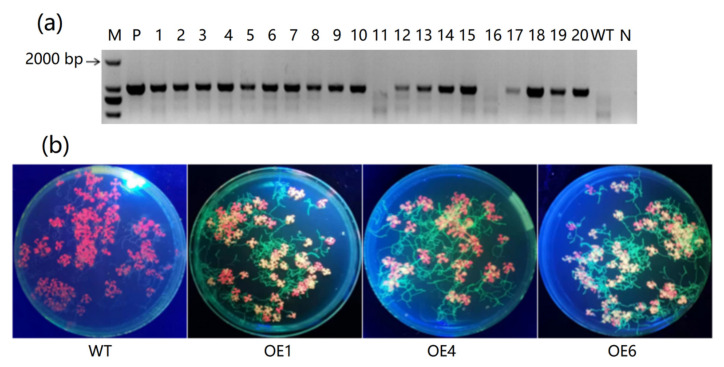
Detection of transgenic *Arabidopsis* heterologously expressing *MeAnn2*. (**a**) PCR detection of the leaves of resistant transgenic *Arabidopsis* seedlings, T1 generation. M: DL2000; P: Positive plasmid control; 1–20: Transgenic *Arabidopsis* lines; WT: Columbia-0 *Arabidopsis*; N: Negative control. (**b**) The fluorescent protein excitation light source irradiation results of some transgenic *Arabidopsis* lines.

To reveal the function of *MeAnn2* in abiotic stress resistance, the pCambia1300-35S-*MeAnn2*:*GFP* vector was transformed into *Arabidopsis* Columbia-0. Twenty independent transgenic lines were obtained, among which the identity of 18 lines had been previously confirmed by PCR analysis (Figure 4a), and these lines were designated as OE1–18. The transcription levels were determined by fluorescence detection, and three lines (OE1, OE4, and OE6) with the different fluorescence intensity were selected for subsequent function analysis (Figure 4b). 

### 2.5. Effect of Different Ca^2+^ Concentrations on Arabidopsis Seedlings

**Figure 5:**
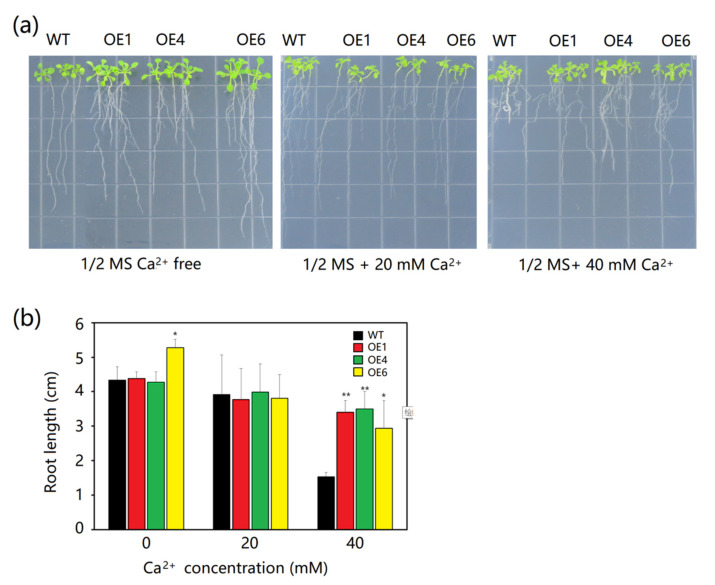
(**a**) Phenotype and (**b**) root length of transgenic *Arabidopsis* overexpressing *MeAnn2* in response to a Ca^2+^ concentration gradient. Error bars indicate ± SD. *, *n* = 3, *p* ≤ 0.05; **, *p* ≤ 0.01.

In terms of root length, no differences were found between the *MeAnn2*-overexpressing *Arabidopsis* lines (OE1, OE4, and OE6) and wild-type *Arabidopsis* (WT) when they were cultured on 1/2 MS medium containing 20 mM Ca^2+^. The growth of the lateral roots and leaves of WT was inhibited on Ca^2+^-free 1/2 MS medium, while the growth of the lateral roots and leaves of MeAnn2 overexpressing lines was promoted. At a higher Ca^2+^ concentration (40 mM), the root length of WT was severely inhibited, with an average root length of about 1.5 cm, while the average root length of transgenic *Arabidopsis* ranged from 2.9 to 3.5 cm. This was significantly longer than that of WT (Figure 5). 

### 2.6. Effects of NaCl Stress on Growth in MeAnn2-Overexpressing Arabidopsis

**Figure 6:**
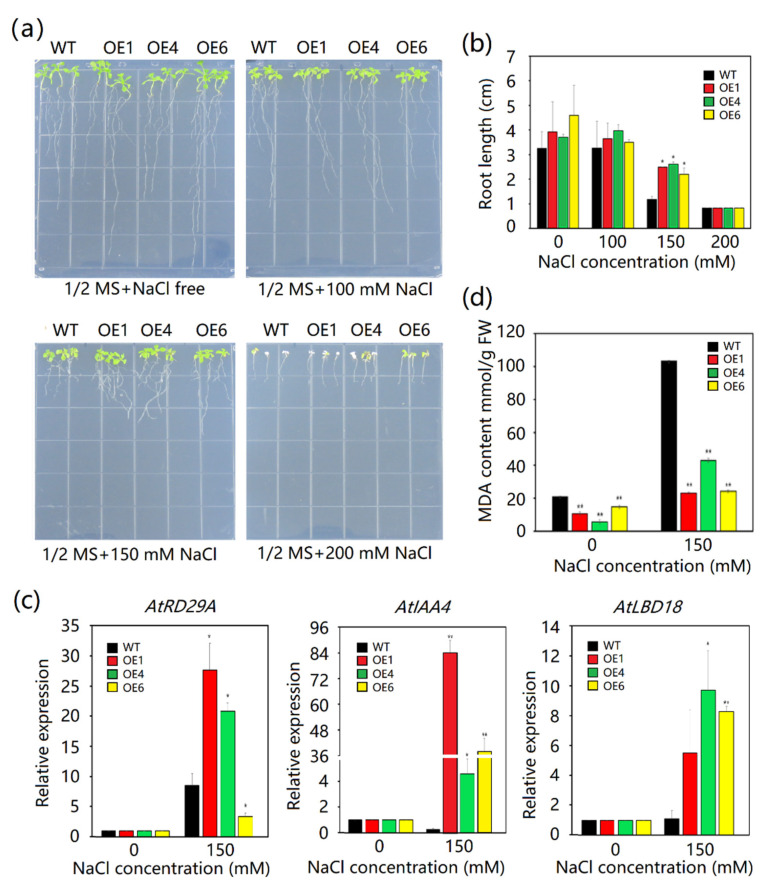
Evaluation of salt resistance in *MeAnn2* OE plants. (**a**) Phenotypes of transgenic *Arabidopsis* under different NaCl concentration gradient stresses. (**b**) Root length of transgenic *Arabidopsis* under different NaCl concentration gradient stresses. (**c**) The expression of stress resistance-related genes under 150 mM NaCl stress. (**d**) MDA content under 150 mM NaCl stress. Error bars indicate ± SD. *, *n* = 3, *p* ≤ 0.05; **, *p* ≤ 0.01.

The phenotypes of the transgenic *Arabidopsis* lines overexpressing *MeAnn2* (OE1, OE4, and OE6) were similar to those of wild-type *Arabidopsis* (WT) on 1/2 MS without NaCl medium. On the medium with 100 and 150 mM NaCl added, the upper parts of the plant seedlings grew similarly between the transgenic lines and the WT, while the roots in the transgenic lines had significantly better development with more lateral roots than in the WT. In the medium containing 200 mM NaCl, growth of both the transgenic and WT *Arabidopsis* was inhibited, and all plants gradually yellowed and died. However, the yellowing speed of the transgenic plants was slightly slower than that of the control (Figure 6a,b).

Gene expression analysis indicated that the expression levels of the stress resistance-related gene *AtRD29B*, and the auxin pathway-related genes *AtLBD18* and *AtIAA4* were significantly increased under 150 mM NaCl stress in the transgenic *Arabidopsis* overexpressing *MeAnn2* (Figure 6c). The MDA levels were significantly lower in the transgenic lines compared with the wild type (Figure 6d).

### 2.7. The Effects of IAA on MeAnn2 Overexpressing Arabidopsis 

**Figure 7:**
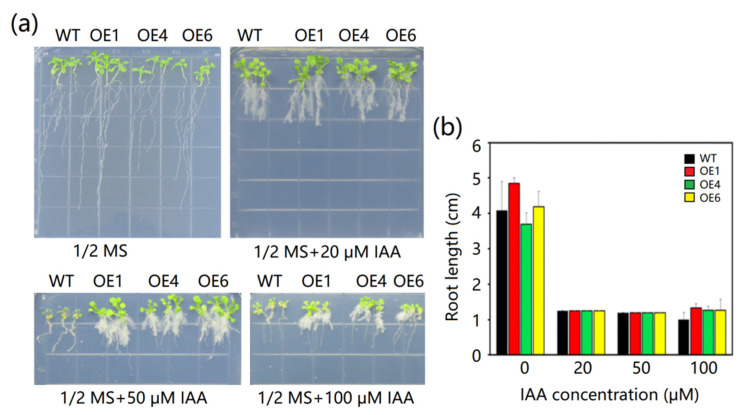
(**a**) Phenotypes and (**b**) root length of transgenic *Arabidopsis* under IAA stress. Error bars indicate ± SD.

To study whether *MeAnn2* directly responds to indole-3-acetic acid (IAA) signals, the growth of transgenic *Arabidopsis* lines overexpressing *MeAnn2* (OE1, OE4, and OE6) was compared with that of control *Arabidopsis* when grown on 1/2 MS medium containing various IAA concentrations for 10 days. The results show that on the medium without IAA, the growth was similar between the transgenic and WT *Arabidopsis*. On the medium with 20 μM IAA, the taproot length of both the transgenic and wild-type *Arabidopsis thaliana* was inhibited, but the lateral roots were developed and the leaves were enlarged. The phenotypes of *MeAnn2*-overexpressing and wild-type *Arabidopsis* showed little difference. On the medium containing 50 and 100 μM IAA, the growth and development of the lateral roots and leaves of wild-type *Arabidopsis* were inhibited, while the transgenic lines had abundant lateral root formation and larger leaves (Figure 7). The above results indicate that the *MeAnn2* gene responds to IAA and participates in the IAA signaling pathway, and its transformation and expression in *Arabidopsis* resulted in reduced sensitivity of the transgenic plants to IAA.

## 3. Discussion

Plant annexins are Ca^2+^ signal receptive proteins and are part of the first step in the signal transduction pathway in addition to playing important roles in plant biological development and responses to adversity and stress [1,5,38,39,40]. *Arabidopsis* annexin mediates the calcium and potassium permeability of the root cell plasma membrane, and participates in an increase in [Ca^2+^]_cyt_ in roots and seedlings in response to hydrogen peroxide [14,16,41]. The *AtAnn1* gene responds to drought, low temperature, and salt stresses and mediates the calcium-dependent system defense of *Arabidopsis* [14,15,42,43]. It is one of the key genes in *Arabidopsis* resistance to stress. In *Arabidopsis*, *AtAnn8* participates in cell death and disease resistance through negative regulation of RPW8.1 [44]. *Capsicum annuum* annexin 24 (Ca32) acts as an ion channel to regulate calcium influx in liposomes [45]. *Populus euphratica* *PeAnn1* promotes the accumulation of cadmium in transgenic *Arabidopsis* [46]. Wheat annexin, *TdAnn6*, participates in resistance to salt and osmotic stress by regulating antioxidant mechanisms [28]. *OsAnn10* is a negative regulator of resistance to osmotic stress in rice (*Oryza sativa*) [47], while *OsAnn1* enhances heat stress tolerance [48]. The cotton (*Gossypium hirsutum*) annexin *GhANN8b* participates in the regulating of Ca^2+^ and Na^+^ fluxes in plant salt tolerance. [29]. Tobacco seedlings heterologously expressing mustard (*Brassica juncea*) *BjAnn1* and *BjAnn2* have enhanced tolerance to salt damage [25,26]. *AtAnn1* is the most studied annexin gene in *Arabidopsis*, and its key role in plant stress resistance has attracted our attention. As a tropical and subtropical plant, cassava has good drought tolerance. The roles of the annexin genes in this plant remain as yet unverified. In this article, we studied the characteristics and functions of the *MeAnn2*, whose protein has 70.9% amino acid sequence similarity with AtAnn1. In Figure 1a, from the protein sequence alignment, it is easy to determine that the third, fifth and seventh green box indicated that Ca^2+^ binding motifs were different in MeAnn2 compared with AtAnn1. In MeAnn2, it is “LHWTL” in the third green box, while in PeAnn1, AtAnn1, BjAnn1, CkAnn1 and StAnn1 the motif is “LLWTL”. In the fifth green box, the motif “KAYS” was the same in MeAnn2 and PeAnn1, but different to “KHYN” in AtAnn1, BjAnn1 and CkAnn1. In the seventh green box, the motif “NKRGT” was the same in MeAnn2, PeAnn1 and CkAnn1, but it was “NKTGT” in AtAnn1 and BjAnn1, “KKLGT” in StAnn1, and “AGMGT” in OsAnn1. Whether the changed amino acid residues in the motifs affect the function of MeAnn2 needs further study. 

The cellular location of genes is closely related to their functions. Most of the known plant annexins are mainly located in the cytoplasm, and a small number are located in the plasma membrane, inner membrane, and nuclear membrane [5]. For example, in *Medicago truncatula, MtAnn1* is located in the nuclear membrane [6]. In the present study, we found that *MeAnn2* was localized in the cell membrane, nucleus and cytoplasm. This indicates that annexins of the same family do not function at exactly the same locations. 

Under normal conditions, the expression of annexins is dynamic in plant tissues and organs during plant growth processes [9,10,11,12,13]. The analysis of tissue-specific expression levels indicated that the *MeAnn2* gene can be detected in all the tested tissues and organs of cassava, but the expression level is highest in the roots, followed by the stems and root tips, and is lowest in the leaves. The expression levels and locations of plant annexins determine their various functions, such as in plant growth, cell secretion, Ca^2+^ metabolism, and regulating abiotic stress. 

Ca^2+^ is the most prevalent second messenger in plants and plays a crucial role in signaling pathways related to many abiotic stresses, such as in the response of plants to salt and in drought tolerance. It has been found that annexin can promote Ca^2+^ influx in transgenic *Arabidopsis* protoplasts, thereby increasing the intracellular Ca^2+^ concentration [14,15,16]. In this study, through the overexpression of *MeAnn2* in *Arabidopsis*, we found that the root system of transgenic *Arabidopsis* was more developed than the wild type under no Ca^2+^ (0 mM) and a high Ca^2+^ concentration (40 mM), indicating that *MeAnn2* can reduce the Ca^2+^ sensitivity of plants and thus maintain plant growth.

There are many reports on the functions of annexins in plant stress resistance. For example, *AtAnn1* overexpression in transgenic plants enhanced resistance to drought stress, while mutants in which *AtAnn1* gene was silenced were more sensitive [43]. In addition, following salt stress, *Arabidopsis AtAnn1* and *AtAnn4* [21], mustard *BjAnn3* and *BjAnn7* [24,25], alfalfa *MsAnn2* [49], and tobacco *NtAnn12* had differential expression [50]. Tobacco seedlings overexpressing *BjAnn1* showed enhanced tolerance to salt stress [25]. Our results preliminarily indicated that *MeAnn2* overexpression in transgenic *Arabidopsis* lines led to improved salt stress tolerance compared to wild *Arabidopsis*. For example, under 150 mM NaCl stress conditions, the transgenic *Arabidopsis* had a longer root length and more lateral roots than WT. Meanwhile, the expression of the stress resistance-related gene *AtRD29B* and lateral root development-related auxin pathway genes *AtIAA4* and *AtLBD18* were meaningfully increased in transgenic *Arabidopsis* compared to the control plants, while the MDA content in transgenic *Arabidopsis* was significantly reduced. The *AtRD29B* is one of the key stress response genes always analyzed in gene function analysis. Its up-regulated expression is usually associated with the tolerance of transgenic plants to salt or other abiotic stresses [35,51,52]. In this study, we found that *AtRD29B* was significantly upregulated by overexpression *MeAnn2* gene in *Arabidopsis* under salt stress. This result indicates that MeAnn2 as a positive regulator mediates plant tolerance to salt stress. 

**Figure 8:**
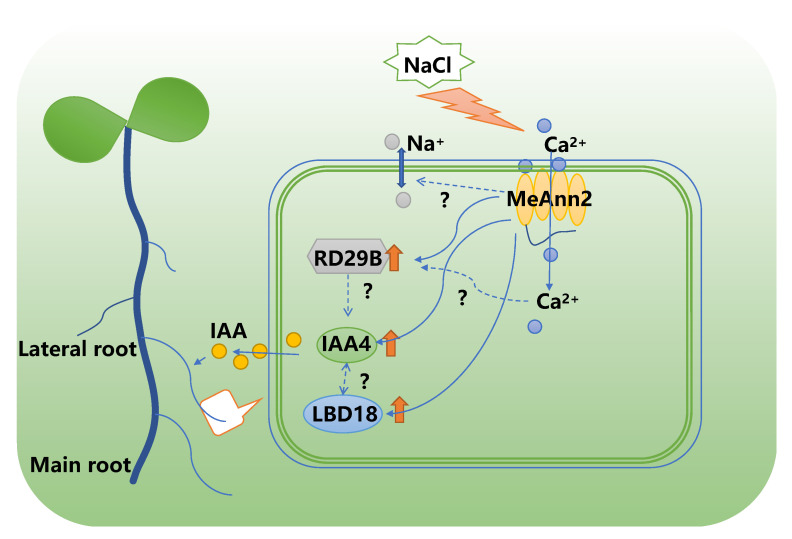
Predicated mechanism of MeAnn2 in improved plant salt tolerance. The orange arrows indicate the gene up-regulated. The question marks indicate the unknown mechanism.

Previous studies have revealed that the elongation of primary roots, development of lateral roots, and changes in root gravity when plants are exposed to salt stress are very important adaptation strategies for plants to survive high levels of salt. Salt stress affects the lateral root number, root development, and root growth direction of plants by regulating the concentration gradient and redistribution of IAA [53]. The previous research indicated that the high expression of *AtLBD18* and *AtIAA4* was related to the salt tolerance of transgenic *Arabidopsis* [35]. In our analysis of transgenic *Arabidopsis* overexpressing *MeAnn2* under salt stress, in which salt tolerance was enhanced compared to WT, it was found that the expression levels of the related genes *AtIAA4* and *AtLBD18* in the auxin signaling pathway were significantly increased. Therefore, we can speculate that the improved salt tolerance of transgenic *Arabidopsis* overexpressing *MeAnn2* may be related to effects on auxin signaling. We then investigated whether *MeAnn2* directly responds to IAA. The results show that, in response to exogenous IAA application, the lateral roots and leaves of transgenic *Arabidopsis* were more developed than those of the wild type, indicating that IAA had a stronger effect on the induction of lateral root formation in transgenic *Arabidopsis*. Above all, through the overexpression of the *MeAnn2* gene in *Arabidopsis*, we confirmed the response function of *MeAnn2* in Ca^2+^, NaCl and IAA. Additionally, *MeAnn2* might improve the salt tolerance of transgenic *Arabidopsis* by improving the expression of the stress-related *RD29B* gene and participating in the response of *IAA4* mediating the auxin signaling pathway, which would promote the LBD18 and IAA4 regulated root growth under salt stress (Figure 8). However, there are still a lot of deep regulatory mechanisms to be studied.

In this study, *MeAnn2* overexpressed transgenic *Arabidopsis* was only compared with WT, but it was better compared with *Arabidopsis* overexpressed *AtAnn1* or *AtAnn1* mutants. Fortunately, previous studies have shown that *AtAnn1* mutants inhibit root growth under salt stress, suggesting that *AtAnn1* is involved in root morphogenesis [15]. Therefore, the overexpression of *MeAnn2* in our study enhanced root growth under salt stress, which indicated that *MeAnn2* in cassava was also involved in root morphogenesis to a certain extent. We analyzed the expression of the *AtAnn1* gene in the *MeAnn2* overexpression transgenic *Arabidopsis* OE1, OE4 and OE6 (Figure S2). To our surprise, we found that endogenous *AtAnn1* expression was inhibited, and the inhibition trend was consistent with the overexpression trend of *MeAnn2*, that is, the more overexpression of *MeAnn2*, the stronger the inhibition degree. This may be due to the high similarity between MeAnn2 and AtAnn1 protein sequences, and thus the high similarity in their functions, leading to functional redundancy when one of the genes is overexpressed.

## 4. Materials and Methods

### 4.1. Total RNA Extraction and PCR Amplification of the MeAnn2 Gene

One-month-old cassava cultivar SC8 seedlings were used as material for total cassava RNA extraction using the RNAprep Pure Plant Total RNA Extraction Kit (TianGen, Beijing, China) according to the manufacturer’s instructions. After detection by 1% gel electrophoresis, the total RNA was first treated to remove the genomic DNA, then transferred into the first strand of cDNA by using the PrimeScript RT reagent kit with gDNA Eraser (TaKaRa, Dalian, China) according to the manufacturer’s instructions. The first strand of synthesized cDNA was used as a template, and Me-Tubulin-F/R was used as upstream and downstream primers to detect the quality of reverse transcription.

To clone the cDNA of *MeAnn2* from cassava, the primer pairs *MeAnn2*-F: GAGTCTACACAGAGCAGAGAGCCT and *MeAnn2*-R: TATTCCCCGTTGAAACCACA, were designed using Primer Premier 5 outside the coding region, and the high-fidelity enzyme Prime STAR HS (Premix) (TaKaRa, Dalian, China) was used to perform the PCR reaction. The amplified product was recovered, directly ligated to the pMD-19T vector using the TA cloning method according to the operating instruction of pMD™19-T Vector Cloning Kit (TaKaRa, Dalian, China), and then transformed into DH-5α competent cells. The positive clones, identified by bacterial liquid PCR using the BcaBEST Sequencing Primers RV-M and M13-47 in the pMD-19T vector, were sent for DNA sequencing.

### 4.2. MeAnn2 Gene Bioinformatics Analysis and Gene Expression

The physical and chemical properties of the MeAnn2 encoded protein were predicted using the ProParam online software (https://web.expasy.org/protparam/, accessed on 10 April 2021). The sequences of annexin genes in *Arabidopsis thaliana*, *Populus euphratica*, *Oryza sativa*, *Solanum tuberosum*, *Cynanchum komarovii*, *Brassica juncea*, and *Manihot esculenta* were obtained from the NCBI database (https://blast.ncbi.nlm.nih.gov/Blast.cgi, accessed on 10 April 2021). The amino acid sequences translated from each annexin gene can be found in the Supplementary Materials (Table S1). The multiple protein alignment was performed using Jalview in Phytozome net. The phylogenetic tree was created using MEGA 7.0 software. The protein sequences were first aligned by the ClustalW in the MEGA 7.0 software with the default parameters. Then, the result was used to construct the phylogeny tree, by using the neighbor-joining statistical method, with the Bootstrap method as the test of phylogeny, and the number of Bootstrap replications was 1000, the model was a Poisson model, and the Gaps/Missing data treatment was Pairwise deletion. The expression level of *MeAnn2* in different organs and tissues was calculated according to the publicly available cassava RNA sequencing data (Bioproject ID PRJNA324539) [54]. The transcriptome data were download from the NCBI web (https://www.ncbi.nlm.nih.gov/bioproject/PRJNA324539, accessed on 10 April 2021). The FPKM data of *MeAnn2* gene expression in different organs and tissues, such as fibrous root, storage root, root apical meristem, stems, stem apical meristem, leaf, petiole, midvein, lateral bud, organized embryogenic structure, and friable embryogenic calli, were selected to analyze and draw the histogram using Excel software.

### 4.3. Subcellular Localization of MeAnn2:GFP

SnapGene software was used to design the primers p1300-*MeAnn2*-F: ACGCGTCGACATGTCTACCCTTATAG (where the *Sal*I restriction site is underlined) and p1300-*MeAnn2*-R: CGCGGATCCACTCAATCCTCCTTATG (where the *BamH*I restriction site is underlined). Using the double-enzyme digestion method, the target gene was inserted into the plant overexpression vector pCAMBIA1300 to construct the pCAMBIA 1300-*MeAnn2*:*GFP* vector. The primer pairs, designed on the pCAMBIA1300, 1300-F: GTTGATACATATGCCCGTCG and 1300-R: CTCGCCCTTGCTCACCAT, were used for the detection and verification of the recombinant vector. The recombinant plasmid and the pCAMBIA1300-*GFP* empty vector were transformed into competent LBA4404 *Agrobacterium* cells, which were injected into tobacco leaves. The MeAnn2:GFP protein localization was observed under a laser confocal microscope (OLYMPUS FV1000) 3–5 days after the injection.

### 4.4. Genetic Transformation and Identification of MeAnn2 Overexpression Transgenic Lines

*Agrobacterium* containing the recombinant pCAMBIA1300-*MeAnn2* overexpression vector was transfected into *Arabidopsis thaliana* Col-0 inflorescences, and the infected mature seeds (T0) were collected and cultured on 1/2 MS (containing 100 mg/L hygromycin) solid medium to screen for positive transformants. The *Arabidopsis* seedlings of various lines capable of rooting on hygromycin-containing medium were identified by leaf PCR with 2× M5 HiPer Superluminal mix with blue dye (Mei5bio, Beijing, China) and the detection primers 1300-F and 1300-R (same in the Section 4.3). A Luyor-3415RG dual-wavelength fluorescent protein excitation light source was used to observe whether the transgenic plants (T1) emitted green fluorescence, and the fluorescence intensity was used to qualitatively detect *MeAnn2* expression. Three transgenic lines with strong green fluorescence were selected for subsequent experiments. To evaluate the expression levels of *MeAnn2* and *AtAnn1* in the transgenic *Arabidopsis* lines, the total RNA was isolated from the transgenic *Arabidopsis* lines OE1, OE4, OE6, and wild-type *Arabidopsis* using the RNAplant Plus reagent (TianGen, Beijing, China) based on the manufacturer’s instructions. Additionally, the RNA first erased the genome DNA and then reversed to cDNA as described in 4.1. SYBR^®^ Premix Ex Taq^TM^ II reagent (TaKaRa, Dalian, China) was used to analyze the expression level of each gene. The *Arabidopsis* actin gene was employed as an internal reference. The 2^−^^ΔΔCt^ method was used to calculate the relative expression level. Each sample of the experiment had three biological replicates, and the calculated results were plotted with Excel. The primer pairs used were as follows: *MeAnn2* (*QMeAnn2*-F: CCCTTGAAGAGGATGTGGCG and *QMeAnn2*-R: TTCACCTCAGCCCC- CTCGTAT); *AtAnn1* (*QAtAnn1*-F: TGAACCCGGTGAGCGTGATG and *QAtAnn1*-R: A-GCAGCTGCGTTGATGTCCT); *AtActin* (*QAtActin*-F: TTCCTCATGCCATCCTCCGTCT- T and *QAtActin*-R: CAGCGATACCTGAGAACATAGTG). 

### 4.5. Transgenic Arabidopsis Stress Treatment

Under aseptic conditions, three T3 generation transgenic *Arabidopsis* lines, OE1, OE4, OE6, along with wild-type Col-0, were grown on 1/2 MS medium for about 6 days. Plants that had two true leaves were then moved onto 1/2 MS medium square plates to undergo different stress treatments and were cultured at 22 °C in 12 h light/12 h dark conditions. Ten days later, the phenotypes and root length were observed. The Ca^2+^ treatments were performed by the inclusion of 0, 20, and 40 mM CaCl_2_ into 1/2 MS medium. The NaCl treatments were performed by inclusion of 0, 100, 150, and 200 mM NaCl into the 1/2 MS medium. The IAA treatments were performed by inclusion of 0, 20, 50 and 100 μM IAA into the 1/2 MS medium. In this experiment, three biological replicates were performed.

### 4.6. Quantitative RT-PCR Analysis of Stress Resistance-Related Genes

Total RNA was isolated from the treated transgenic *Arabidopsis* lines OE1, OE4, OE6, and wild-type *Arabidopsis* using the RNAplant Plus reagent (TianGen, Beijing, China) based on the manufacturer’s instructions. TaKaRa SYBR^®^ Premix Ex Taq^TM^ II reagent (TaKaRa, Dalian, China) was used to analyze the expression level of related stress resistance genes in *Arabidopsis*. The *Arabidopsis* actin gene was employed as an internal reference. The 2^−^^ΔΔCt^ method was used to calculate the relative expression level. Each sample of the experiment had three biological replicates, and the calculated results were plotted with Excel. The detected genes and their specific primers are as follows: *AtIAA4* (*QAtIAA4*-F: GCAGAGGAGGCAATGAGTAGTG and *QAtIAA4*-R: GAGCATCCAGTCACCATCTTTG), *AtRD29B* (*QAtRD29B*-F: ATTCACCATCCAG AAGAAGAGCATC and Q*AtRD29B*-R: ACTTCTGGGTCTTGCTCGTCA), *AtLBD18* (*QAtLBD18*-F: GTCGCTCACATCTTTGCTCTTC and *QAtLBD18*-R: GTCGCTCACATC TTTGCTCTTC), *AtActin* (*QAtActin*-F: TTCCTCATGCCATCCTCCGTCTT and Q*AtActin*-R: CAGCGATACCTGAGAACATAGTG) [35]. 

### 4.7. Statistical Analysis

The data are presented as the mean ± SD. The significance of the data was evaluated by one-way ANOVA with Duncan’s multiple range test. *p* ≤ 0.05 and *p* ≤ 0.01 were set as the significance levels.

## 5. Conclusions

In summary, the function of *MeAnn2*, the cassava homolog of *AtAnn1*, was studied in the context of stress tolerance. The overexpression of *MeAnn2* reduced the Ca^2+^, NaCl, and IAA sensitivity of transgenic *Arabidopsis*. The enhanced salt tolerance of transgenic *Arabidopsis* was accompanied by the upregulation of the stress tolerance-related gene *AtRD29B* and the auxin signaling pathway-related genes *AtIAA4* and *AtLBD18*, as well as a reduction in MDA content. These results suggest that *MeAnn2* might improve the salt tolerance of these plants through the auxin signaling pathway. 

## Supplementary Materials

The following are available online at https://www.mdpi.com/article/10.3390/plants10050941/s1, Table S1. Amino acid sequences of annexins from different plants. Figure S1. Cloning and sequence analysis of MeAnn2 cDNA from SC8 cassava. Figure S2. qRT-PCR analysis of the expression of (a) *MeAnn2* and (b) *AtAnn1* in transgenic *Arabidopsis* lines.
