# Supplementary material for: Overexpression of Cassava MeAnn2 Enhances the Salt and IAA Tolerance of Transgenic Arabidopsis
Source: Plants (Basel). 2021 May 8;10(5):941. doi: 10.3390/plants10050941 (PMC8150822; doi:10.3390/plants10050941)
Supplement: Supplementary file 1 [file plants-10-00941-s001.zip › Figure S1-S2.pdf]

(a)

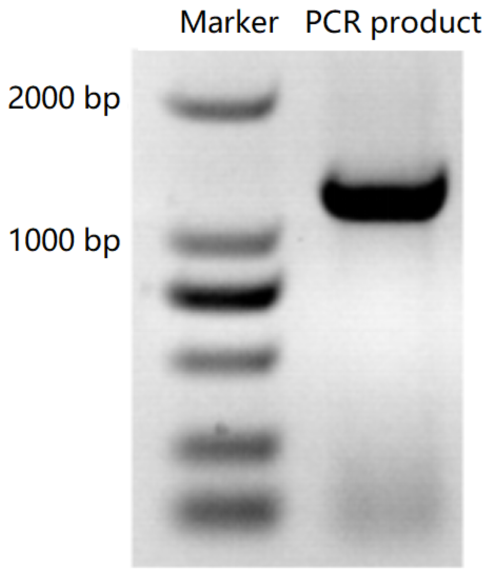

(b)

Primer MeAnn2-F

GAGTCTACACAGAGCAGAGAGCCTGAGGGAGTGTGAGCTTCGCAGATCGTTGATAG
AAAAAAGACACAGATCCAAAGCACTCGACAATGTCTACCCTTATAGTTCCTCATAC
AGTTCCACCTGTTTCCGATGACTGTGAGCAGCTCAGAAAGGCTTTTTCAGGATGGG
GGACAAACGAGGGCTTGATCATATCCGTATTGGCTCACAGGAATGCTGCTCAACGC
AAGCTGATCAGAGAGACGTATTATGAGGCCTACGGAGAAGATCTCCTCAAGGAATT
GGATAGGGAACTTTCAAATGATTTTGAGAGAGTGGTGCTGCACTGGACACTTGGTC
CTTCTGAACGCGATGCAGTTTTAGCTAATGAGGCAGCTAAGAAATGGACATCAAGC
AATCAGGTTCTTATGGAAATTGCCTGCACTAGGTCCTCAAATGAACTGCTTCATGT
AAGACAGGCATATCATGCTCGCTACAAGAAGTCCCTTGAAGAGGATGTGGCGCAGC
ATACAACTGGGGACTTCCGCAAGCTTTTGTTTCCTCTTGTGTGCTCATACCGATAC
GAGGGGGCTGAGGTGAACATGACCCTCGCAAAAACAGAGGCTAAGTTGATCCACGA
GAAAATCTCAAAGAAGGCTTATAGTGATGAGGATATCATCAGGGTTATCGCTACCA
GGAGCAAAGCGCAGATTAATGCTACCGTGAATGAGTACAAAAATGCGTTTGGCAAT
GATATCAACAAGGACTTGAAGGCTGACCCCAAGGATGAATTCCTCTCGTTGTTGAG
GGCCACAGTGAAGTGCTTGACACGCCCAGATAAGTATTTTGAGAAGGTTCTTCGCC
TGGCTATCAACAAAAGAGGAACAGATGAAGGAGCTCTGACTAGAGTTGTGACTACT
AGGGCGGAGGTTGACATGAAGATTATAAAAGATGAGTACCAGCAAAGGAACAGTAT
TCCTTTGGAGACTGCCATTGCCAAGGACACCCATGGGGATTATGAAGATATGCTTC
TGGCGCTGATTGGGCATAAGGAGGATTGAGCTGTTAATCTGTCTCAAGGGGCATTA
TATCCATGAAGGTTGTTGTGCGGCTTGTTGCATGTGGTTTCAACGGGGAATA

Primer MeAnn2-R

Figure S1. Cloning and sequence analysis of MeAnn2 cDNA from SC8 cassava. (a), Gel analysis of PCR product of MeAnn2 cDNA cloning from SC8 cassava. (b), Sequence of the *MeAnn2* cDNA cloning from SC8 cassava. The underline indicated the primer regions. The blue

characters indicated the coding sequences of *MeAnn2* (951 bp).

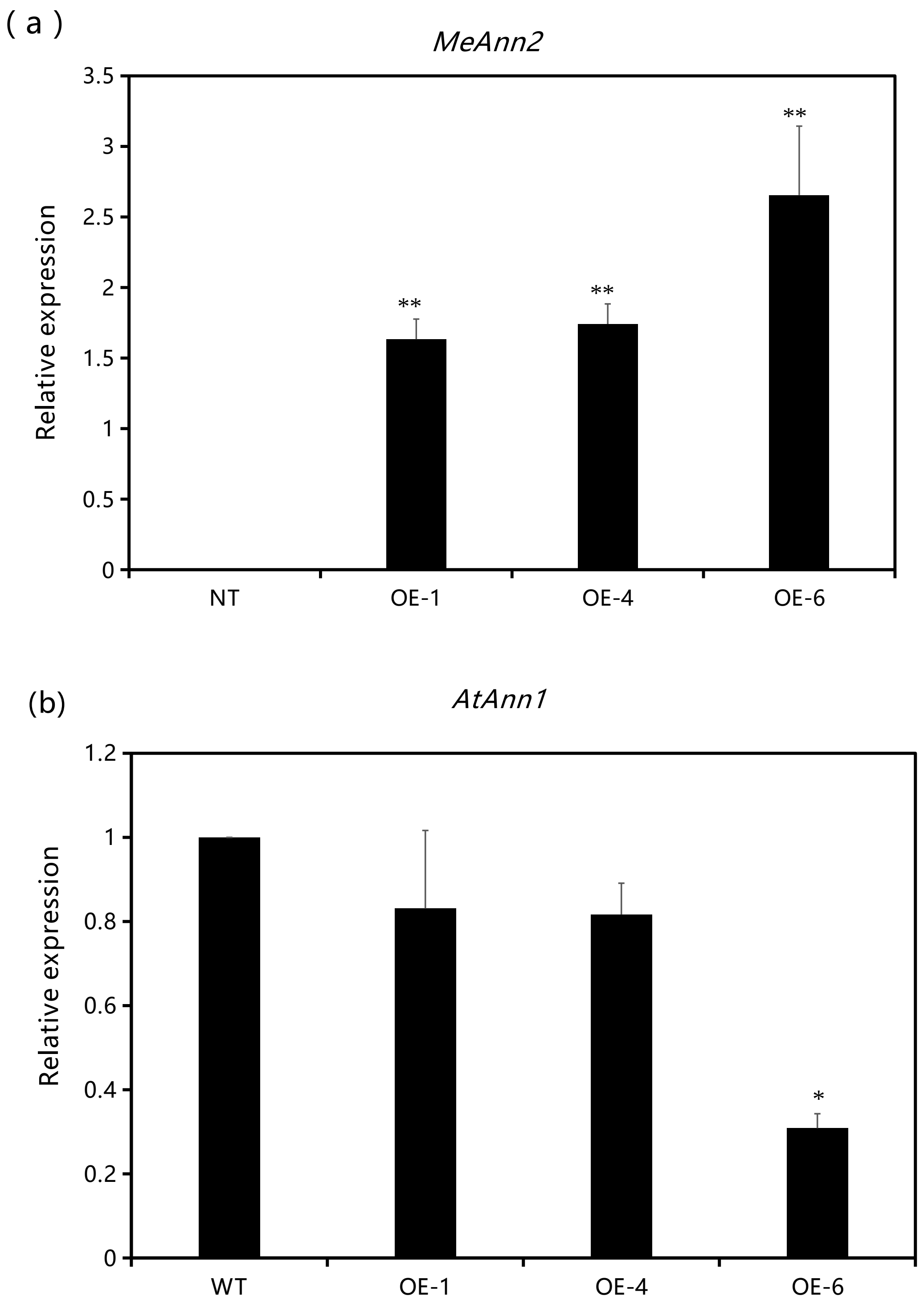

Figure S2. qRT-PCR analysis of the expression of (a) *MeAnn2* and (b) *AtAnn1* in transgenic Arabidopsis lines. Error bars indicate ± SD. *, n=3, P ≤ 0.05; **, P ≤ 0.01.
